# Sphingosine kills Mycobacteria and suppresses mycobacterial lung infections

**DOI:** 10.1007/s00109-025-02534-z

**Published:** 2025-03-28

**Authors:** Yuqing Wu, Fabian Schnitker, Yongjie Liu, Simone Keitsch, Federico Caicci, Fabian Schumacher, Andrea Riehle, Barbara Pollmeier, Jan Kehrmann, Burkhard Kleuser, Markus Kamler, Ildiko Szabo, Heike Grassmé, Erich Gulbins

**Affiliations:** 1 Department of Molecular Biology, Institute of Molecular Biology, University Hospital Essen, University Duisburg-Essen, Essen, Germany; 2 Department of Thoracic and Cardiovascular Surgery, University Hospital Essen, University Duisburg-Essen, Thoracic Transplantation, West German Heart and Vascular Center, Essen, Germany; 3 Department of Biology, University of Padova, Padua, Italy; 4 Institute of Pharmacy, Department of Pharmacology and Toxicology, Freie Universität Berlin, Berlin, Germany; 5 Institute of Medical Microbiology, University Hospital Essen, University of Duisburg, Essen, Germany; 6 Dept. of Surgery, College of Medicine, University of Cincinnati, Cincinnati, OH USA; 7 Present Address: Shanghai Institute of Immunology, School of Medicine, Shanghai Jiao Tong University, Shanghai, China

**Keywords:** Sphingosine, Mycobacteria, Pneumonia, Macrophages, Sphingolipids, Acid ceramidase

## Abstract

**Abstract:**

Tuberculous mycobacterial infections pose a substantial global health burden because of their prevalence and multi-drug resistance. The current approach to tackling these infections primarily involves developing new antibiotics or combining existing ones, an approach that often proves ineffective in the specific targeting of mycobacteria.

We investigated the effect of sphingosine on tuberculous Mycobacteria in vitro and mycobacterial infections in vivo to test whether sphingosine could potentially be used as a novel drug against tuberculosis. Sphingosine inhibited mycobacterial growth and eradicated mycobacteria in vitro. Mechanistically, sphingosine increased bacterial membrane permeability and induced marked changes on the bacterial plasma membrane evidenced by electron microscopy studies. Administration of sphingosine in a mouse model of pulmonary infection with Bacillus Calmette–Guérin (BCG) greatly reduced the number of bacteria in the lung and prevented pulmonary inflammation. Furthermore, infection of ex vivo human lung tissue samples with BCG and treatment with sphingosine showed that sphingosine also kills BCG in human bronchi. Our findings suggest that sphingosine may be a potential therapeutic intervention against mycobacterial infections.

**Key messages:**

- Sphingosine inhibits mycobacterial growth in vitro.
- Sphingosine disrupts bacterial membrane integrity.
- Sphingosine reduces bacterial load in mouse pulmonary infection model.
- Sphingosine eradicates mycobacteria in human bronchi ex vivo.

**Supplementary Information:**

The online version contains supplementary material available at 10.1007/s00109-025-02534-z.

## Introduction

Mycobacterial infections, particularly those caused by *M. tuberculosis*, the pathogen responsible for tuberculosis (TB), have become a reemerging global threat because of the bacterium’s multi-drug resistance. Before the coronavirus (COVID-19) pandemic, TB was the leading cause of death from a single infectious agent, ranking even above HIV/AIDS. According to the Global Tuberculosis Report published by the World Health Organization (WHO), an estimated 23% of the world's population has latent TB, which is asymptomatic, and in 2022 nearly 1.4 million people died of TB.

In pursuing effective treatments, current strategies mainly involve developing new antibiotics or combining existing ones. However, recent research has shed light on the crucial role of sphingolipids and enzymes involved in sphingolipid metabolism, such as neutral sphingomyelinase, acid sphingomyelinase, and sphingosine kinase 1, which have been implicated in the modulation of mycobacterial infections[1–6]. For instance, previous studies have demonstrated the role of neutral sphingomyelinase in regulating the host response to *M. bovis* (BCG) infection[7–9]. Additionally, sphingomyelin biosynthesis is essential for the phagocytosis of *M. tuberculosis* by host cells[10]. More recently, it has been shown that the NPC1 molecule, which is involved in lysosomal sphingolipid metabolism, mediates phagolysosome fusion during *M. tuberculosis* infection in macrophages[11]. These findings collectively highlight the importance of sphingolipids in the context of mycobacterial infections.

Previous studies have demonstrated the antimicrobial properties of sphingosine, a sphingoid base. These studies have highlighted the direct antimicrobial effects of sphingosine against a diverse range of bacteria, including both Gram-positive and Gram-negative species such as *Pseudomonas aeruginosa*[12–16], *Staphylococcus aureus*[16–18], *Acinetobacter baumanii*[15]*, Haemophilus influenzae*[19]*, Staphylococcus epidermidis*[20]*, M. abscessus*[21], and *Escherichia coli*[12]*.* Our group has further demonstrated that sphingosine exerts its antibacterial activity by directly targeting bacterial membranes, leading to an increase in membrane permeabilization and cell death[12].

Mtb and BCG are distinct from other bacteria, due to their unique capsule, which is structurally different from the outer and inner membranes found in most other bacteria. Notably, Mtb and BCG possess a mycolic acid outer membrane, an arabinogalactan layer, glycopeptidolipids, trehalose-6,6-dimycolate, trehalose monomycolate, trehalose polyphleates, and phosphatidyl-myo-inositol dimannoside, among other specialized features of mycobacteria[22]. In addition, the metabolic rate and growth of Mtb and BCG are much slower than most other bacteria, including *M. abscessus*. In comparison to tuberculous mycobacteria, *M. abscessus* is less virulent and primarily affects individuals with cystic fibrosis and bronchiectasis, contrasting to *M. tuberculosis,* a globally prevalent pathogen.

Thus, previous data on the bactericidal effect of sphingosine on bacteria cannot be simply transferred to tuberculous mycobacteria and it is important to test whether sphingosine also targets classic Mycobacteria such as BCG and *M. tuberculosis *in vitro and, more importantly, in vivo in the lung and after infection of human tissues. Both of these activities would be central to the development of sphingosine as a drug against mycobacterial infections.

Here, we investigated the effect of sphingosine on Mycobacteria and mycobacterial infections. We found that sphingosine plays an important role in protecting mice and human tissues from mycobacterial infections. Mechanistically, we found that sphingosine mediates membrane permeabilization of *M. bovis* and *M. tuberculosis* and marked changes of the membrane organization in mycobacteria. To gain a more comprehensive understanding of the effect and mechanism of sphingosine during mycobacterial infections, we used diverse infection systems, including macrophages, mice, and human samples, both ex vivo and in vivo, to evaluate the function of sphingosine in the context of mycobacterial infection.

## Results

### Sphingosine kills slow- and fast-growing mycobacteria in vitro

**Fig. 1:**
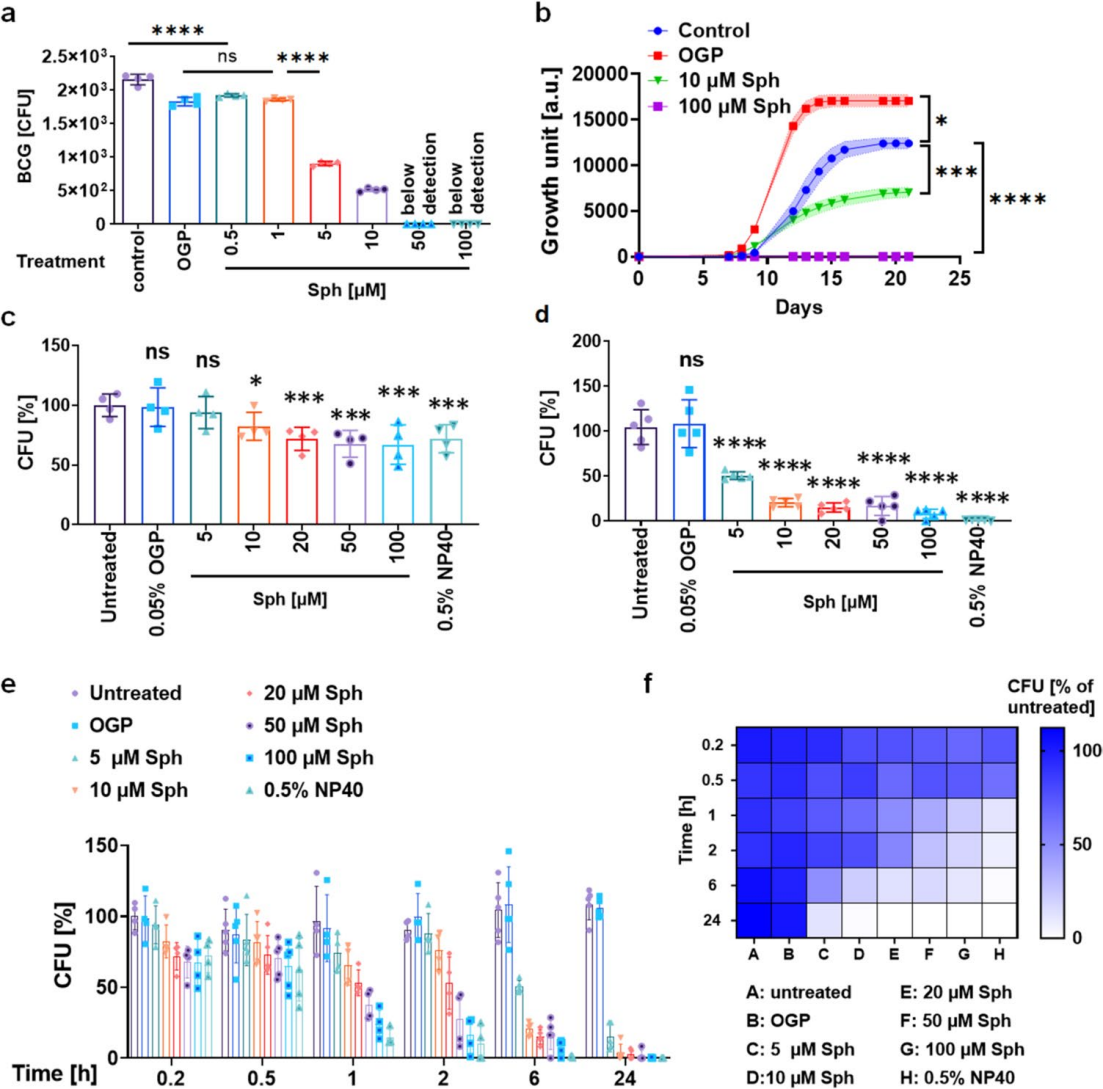
Sphingosine kills slow- and fast-growing mycobacteria in vitro. (**a**) Bacillus Calmette–Guérin (BCG) was treated with sphingosine at the indicated concentrations for 24 h. Bacteria were plated on agar plates, and colony-forming units (CFUs) were counted as a measurement of the survival of BCG after sphingosine treatment. Shown are mean ± SD, n = 4. *p < 0.05, **p < 0.01, ***p < 0.001, ****p < 0.0001, one-way ANOVA. OGP, n-Octyl-β-D-glucopyranoside; Sph, sphingosine. (**b**) *Mycobacterium tuberculosis* was treated with sphingosine at the indicated concentrations for 21 days. Bacterial relative growth counts were measured every 2 to 3 days. Shown are means ± SD, n = 4. *p < 0.05, **p < 0.01, ***p < 0.001, ****p < 0.0001, one-way ANOVA. (**c-f**) *Mycobacterium smegmatis* was treated with sphingosine at the indicated concentration for 10 min (**c**) or 6 h (**d**) or for the indicated times (**e–f**). Bacteria were plated on agar plates, and colony-forming units (CFUs) were counted as a measurement of the survival of the bacteria after sphingosine treatment. NP40, nonyl phenoxypolyethoxylethanol. Panel f shows the heatmap of the CFU result. Shown are means ± SD, *n* = 4. ***p < 0.001, one-way ANOVA

To investigate whether sphingosine kills mycobacteria, we exposed BCG to various concentrations of sphingosine for 24 h and assessed the remaining bacterial colony-forming units (CFUs). Our findings demonstrate that sphingosine exerts a direct and dose-dependent killing effect on BCG in vitro: 5 µM sphingosine reduces bacterial CFUs by 50% (Fig. 1a).

We then evaluated the effect of sphingosine on *M. tuberculosis* strain ATCC 29274, a pathogenic mycobacterial strain causing human tuberculosis, and also a slow growing mycobacterium. Sphingosine or buffer-control was added to the bacterial culture on day 0, and the growth was monitored for 21 days. The results show that sphingosine inhibited the growth of *M. tuberculosis* at a concentration as low as 10 µM and completely eradicated *M. tuberculosis* at 100 µM (Fig. 1b). When we added the solvent of sphingosine, octyl-glucopyranoside (OGP), to the bacteria at the same concentration as that used in the samples treated with sphingosine, it even promoted bacterial growth (Fig. 1b), probably because it served as a carbon source.

To assess the effect of sphingosine on other mycobacterial species, we tested *M. smegmatis*, a fast-growing mycobacterium with a cell wall structure similar to that of *M. tuberculosis* (Fig. 1c-f)*.* Treatment with 20 µM sphingosine killed *M. smegmatis* as early as 10 min after treatment (Fig. 1c). Furthermore, 5 µM sphingosine killed 50% of bacteria after 6 h of treatment (Fig. 1d). Thus, sphingosine also exhibited time- and dose-dependent killing of *M. smegmatis* (Fig. 1e-f).

Controls indicate that octyl-glucopyranoside alone and other lipids such as sphingosine-1-phosphate (S1P) in octyl-glucopyranoside did not kill mycobacteria (Fig. S1).

Collectively, these results indicate that sphingosine has a direct killing effect on both slow- and fast-growing mycobacteria.

### Sphingosine rapidly induces permeabilization of mycobacterial membranes

**Fig. 2:**
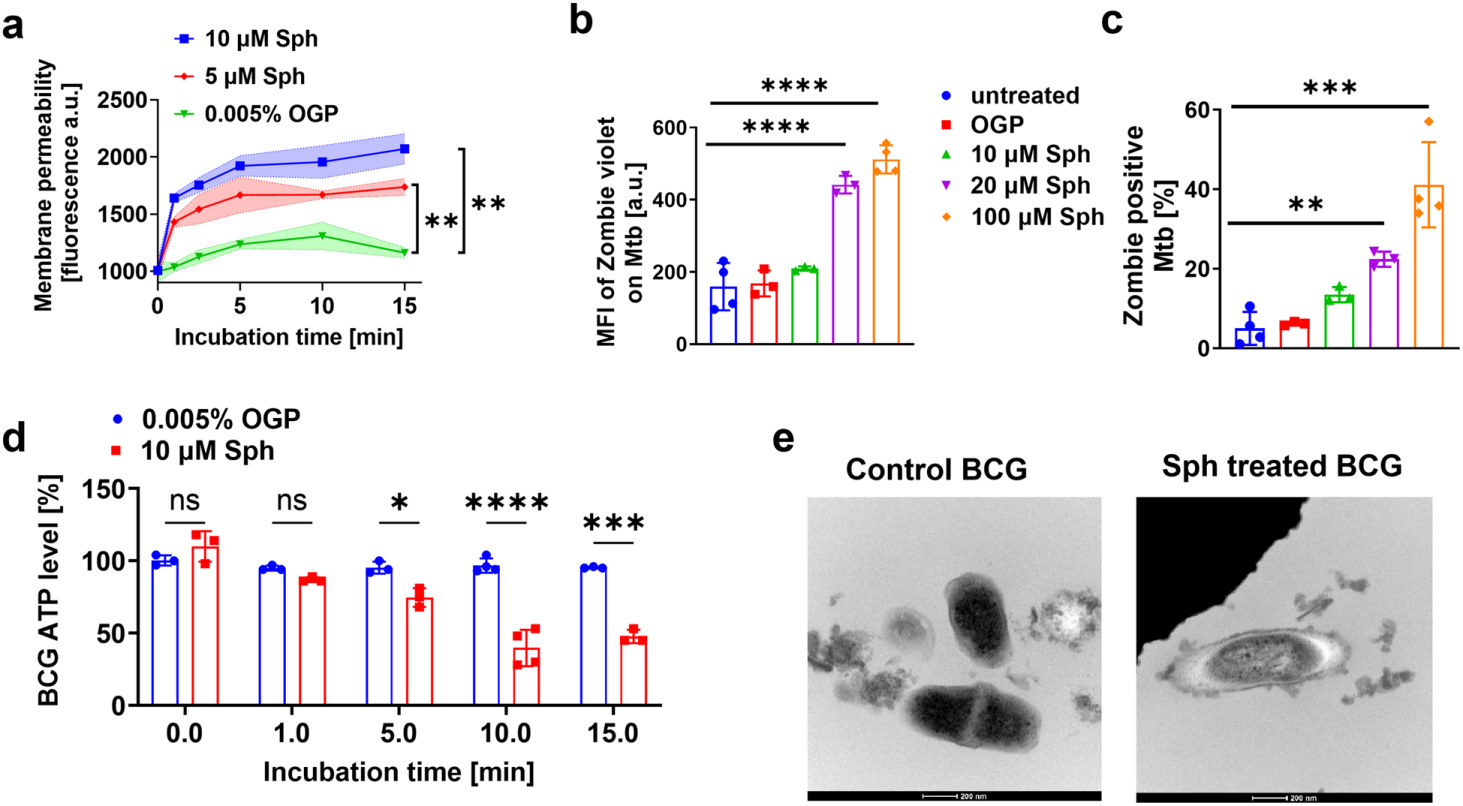
Sphingosine regulates the metabolic profile of mycobacteria and rapidly increases the permeabilization of slow- and fast-growing mycobacterial membranes. (**a**) BCG was incubated with 100 nM TO-PRO-3 iodide. Bacteria were then treated with 5 µM or 10 µM sphingosine (Sph) or the corresponding concentration of octyl-glucopyranoside (OGP), the solvent of sphingosine. The bacteria were analyzed by flow cytometry. Fluorescence intensity is given in arbitrary units (a.u.). (**b-d**) *Mycobacterium tuberculosis* (*Mtb*) was left untreated or incubated with OGP or sphingosine at various concentrations for 10 min and stained with zombie violet. Samples were analyzed by flow cytometry. Given is the (**b**) median fluorescence intensity (MFI) of samples containing zombie violet-positive Mtb and (**c**) the percentage of zombie violet-positive Mtb of 100,000 events. (**d**) The release of ATP from BCG after the indicated time of treatment was measured with the BacTiter-Glo reagent. Fluorescence intensity is given in arbitrary units (a.u.). (**e**) Transmission electron microscopy analysis of untreated BCG (left) and 30 min (right) shows rapid destruction of the bacteria by sphingosine. Shown are the means ± SD or representative results of 3 to 4 independent studies; *p < 0.05, **p < 0.01, ANOVA

To gain insights into the mode of action of sphingosine on mycobacteria, we performed membrane permeability assays. Previous studies have indicated that sphingosine can induce permeabilization of the bacterial plasma membrane and a loss of intracellular adenosine triphosphate (ATP), leading to the killing of other bacteria such as *P. aeruginosa* or *S. aureus*[12]. We incubated BCG with sphingosine and measured membrane permeability by staining the bacteria with TO-PRO-3 iodide. Viable cells are impermeable to TO-PRO 3, which binds to chromosomes after an increase in membrane permeability. The results showed that incubation of BCG with sphingosine rapidly increases bacterial permeability (Fig. 2a).

We then determined membrane permeability in *M. tuberculosis* by using fixable zombie violet, a dye that stains only cells with permeabilized membranes. The results demonstrated that sphingosine treatment leads to a massive increase of *M. tuberculosis* membrane permeability, as evidenced by higher intensity of zombie violet and a higher percentage of zombie-positive *M. tuberculosis* (Fig. 2b,c). These findings suggest that sphingosine targets the mycobacterial membrane, leading to loss of membrane integrity and subsequent bacterial death.

To confirm the rapid and lethal effect of sphingosine on mycobacteria, we measured the release of ATP into the medium after incubation of BCG with sphingosine. The results showed a significant increase in the release of ATP from BCG into the medium (Fig. 2d) upon treatment with sphingosine, whereas OGP, the solvent control of sphingosine, had no effect.

To visualize changes on BCG induced by sphingosine, we performed transmission electron microscopy to investigate the structural changes in BCG caused by sphingosine treatment. In particular, we observed a rolling and a blebbing of the plasma membrane already 10 min after addition of sphingosine (Fig. 2e). At 30 min after the addition of 10 µM sphingosine the bacteria also showed a loss of electron dense material in the cytoplasm (Fig. 2e), a finding further confirming the bactericidal effect of sphingosine on mycobacteria.

### Sphingosine amplifies the killing of BCG by macrophages

**Fig. 3:**
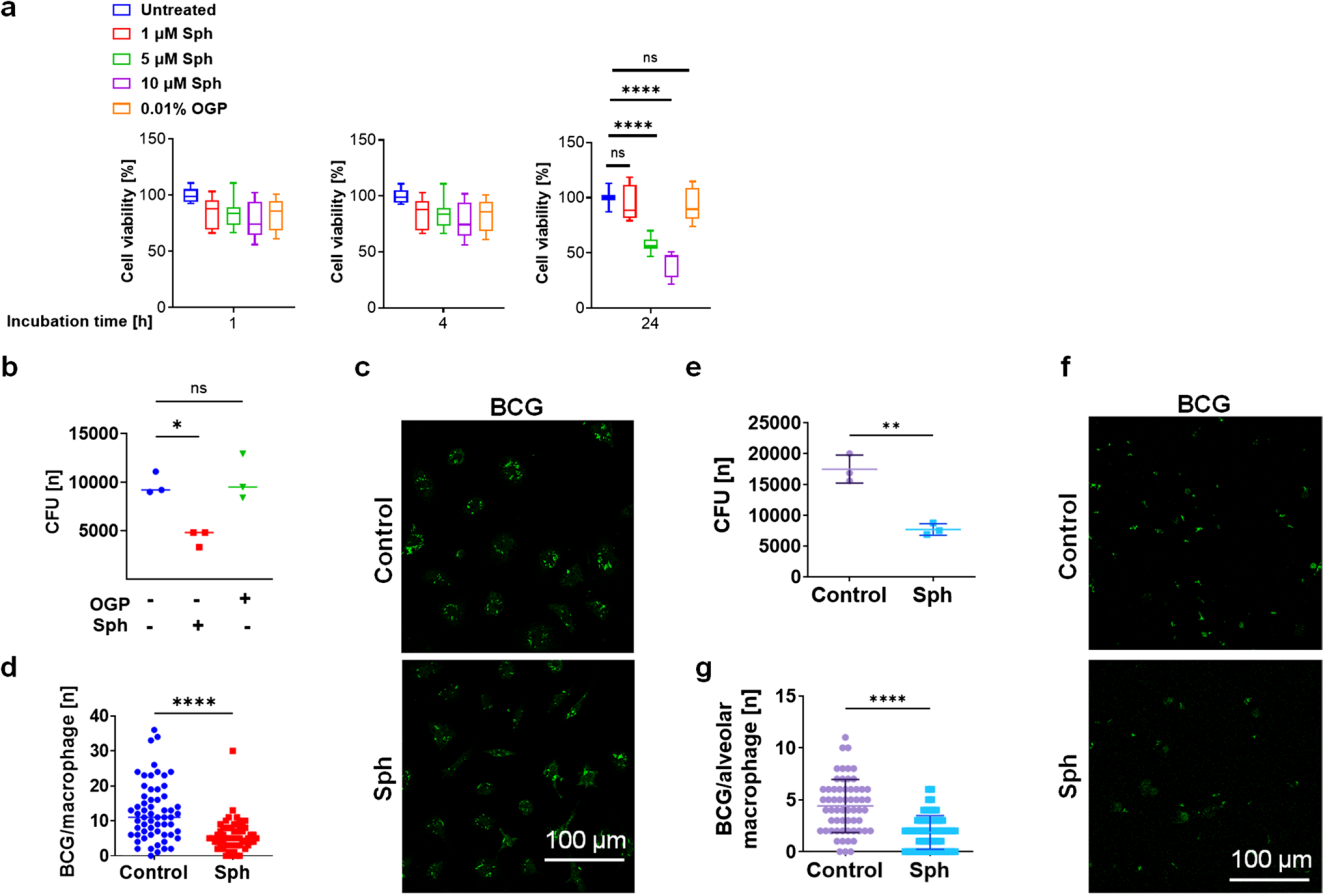
Sphingosine reduces mycobacterial burden in macrophages. (**a**) Bone marrow–derived macrophages (10^4^.) were cultured in 96-well plates and treated for the indicated times with 1 µM (red), 5 µM (green), and 10 µM (purple) sphingosine or 0.01% octylglucopyranoside (OGP) (orange), the solvent of sphingosine. Cells were washed and developed with the MTS kit (Promega) to determine cell viability, n = 3. ****p < 0.0001, one-way ANOVA. (**b-d**) Bone marrow–derived macrophages were isolated, infected for 24 h with BCG expressing GFP, and concomitantly either left untreated or treated with sphingosine or its solvent OGP as controls. (**b**) Cells were lysed, samples were plated, and bacterial CFUs were counted. Panel (**c**) shows representative microscopy studies from 3 independent experiments. Bacteria are indicated by green fluorescence. (**d**) The microscopy studies were quantified by counting the green-labeled bacteria. Shown are the means ± SD of 3 independent experiments, one-way ANOVA. *p < 0.05, ***p < 0.001 ****p < 0.0001, one-way ANOVA. (**e–g**) Alveolar macrophages were isolated, infected for 24 h with BCG, and concomitantly either left untreated or treated with sphingosine or its diluent OGP as controls. (**e**) CFU assays and (**f**) microscopy studies were performed as above. (**g**) The number of GFP-BCG in infected cells was microscopically quantified by counting GFP-expressing BCGs with a 100 × lens. Shown are the mean ± SD or representative results of 3 independent experiments, *t*-test. **p < 0.01, ****p < 0.0001

To test whether sphingosine also affects the killing of mycobacteria upon infection of mammalian cells with the pathogens, we first excluded the possibility that sphingosine impairs the viability of macrophages by treating them with various concentrations of sphingosine. The results showed that 1, 5, or 10 µM sphingosine did not affect the viability of macrophages for at least 4 h. Furthermore, the viability was not affected by 1 µM sphingosine for at least 24 h of sphingosine treatment, whereas higher doses led to impaired viability of macrophages in vitro after a 24-h treatment (Fig. 3a).

To investigate the effect of sphingosine on BCG-infected bone marrow–derived macrophages (BMDMs), we infected cells with BCG and subsequently treated them with 1 µM sphingosine starting 240 min after infection and for a duration of 24 h (Fig. 3b-d). Notably, the incubation of BCG-infected BMDMs with 1 µM sphingosine for the 24-h period led to a significant reduction in pathogen load (Fig. 3b). To further visualize the effects, we performed confocal microscopy studies on macrophages and infected macrophages either untreated or treated with sphingosine. After 24 h of infection, the untreated macrophages exhibited a considerably higher number of bacteria than did the sphingosine-treated macrophages (Fig. 3c, d). These observations provide compelling evidence of the efficacy of sphingosine in limiting bacterial growth.

Next, we investigated whether treatment with sphingosine also reduces infection of freshly isolated murine alveolar macrophages with BCG (Fig. 3e-g). The results confirmed the studies on BMDMs and showed that the treatment of macrophages with 1 µM sphingosine greatly facilitates the elimination of BCG by macrophages (Fig. 3e-g).

### Sphingosine reduces bacterial burden in acute pulmonary infection in vivo

**Fig. 4:**
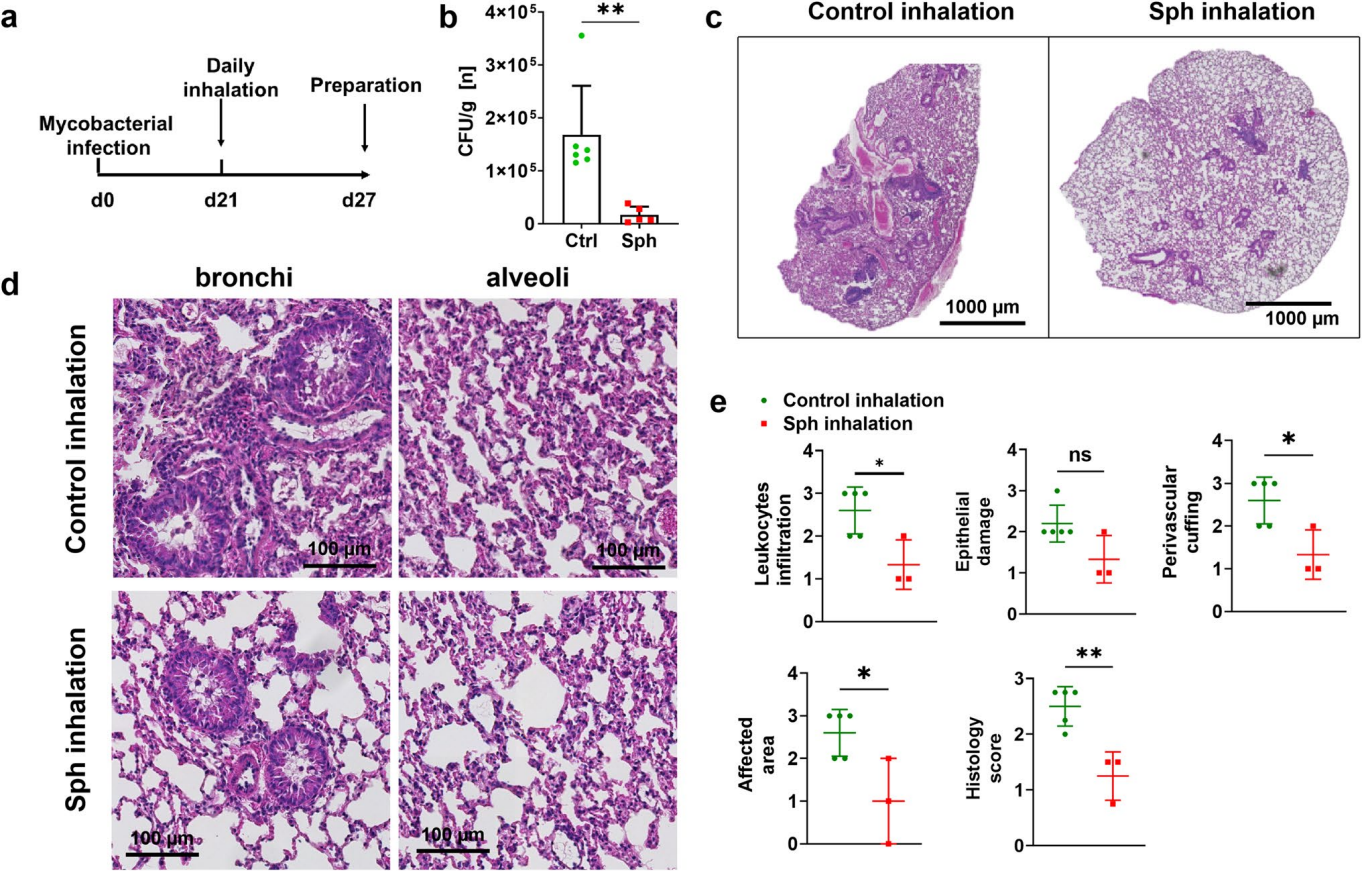
Sphingosine inhalation reduces mycobacteria numbers upon acute pulmonary infection in vivo. (**a**) Timeline for treatment of BCG-infected mice with inhalation after infection. (**b**) Wild-type mice were intranasally infected with BCG for 3 weeks and then inhaled for 7 days with sphingosine or 0.125% octylglucopyranoside, the solvent of sphingosine. The mean ± SD of CFU of each lung is shown, n = 2–10, **p < 0.01, ****p < 0.0001, one-way ANOVA. (**c**) Mice were infected and treated as above, and lung tissues were stained with hematoxylin and eosin and visualized with a 40 × lens. (**d**) The infected and untreated lung showed severe denuding bronchiolitis and debris in the airways, with alterations milder in mice inhaling sphingosine after BCG infection. Shown are representative figures from each of the 3 independent experiments. (**e**) Tissues from control inhaled mice (green) or sphingosine-inhaled mice (red) were scored according to the criteria listed in Table 1, *n* = 3–5, t-test

This finding, taken together with our in vitro findings showing that sphingosine has a direct effect on BCG, led us to assume that sphingosine exerts a direct bactericidal effect in vivo. Therefore, to assess the direct effect of sphingosine in lungs infected with mycobacteria, we pulmonarily infected wild-type mice with BCG for 3 weeks. At this time the bacteria should remain planktonic, similar to active tuberculosis, since a chronic infection has not developed yet. This infection induces in mice a phenotype very similar to that of human infection with *M. tuberculosis* [23]. Starting 2 weeks after infection, we administered either NaCl (containing OGP control) or sphingosine via inhalation for 1 week (Fig. 4a). We then determined whether sphingosine, applied via inhalation, affects BCG in the lung of the mice.

The results showed that sphingosine exerts a remarkable effect on the number of BCG in the lung. Compared to untreated mice or those treated with solvent OGP by inhalation, mice treated with sphingosine by inhalation exhibited an approximately 10- to 50-fold reduction in BCG counts (Fig. 4b). The beneficial effect of sphingosine on BCG infections in vivo was further confirmed by hematoxylin and eosin staining of lung tissue sections (Fig. 4c): The airways of sphingosine-treated mice exhibited no inflammatory cells or debris and an influx of mononuclear cells (Fig. 4d). To quantify this effect, we evaluated various parameters, such as leukocyte infiltration, epithelial cell hyperplasia and damage, perivascular and peribronchiolar cuffing, and the area involved in the infection (Fig. 4e and Table 1).

**Table 1:** Histology score criteria

	Grade 0	Grade 1	Grade 2	Grade 3
Leukocyte infiltration	occasional infiltration of single leukocytes visible	Mild leukocyte infiltration focal infiltration of leukocytes	Moderate coalescing leukocytes individual loci could not be distinguised	Severe infiltration of leukocytes throughout the tissue
Epithelial cell hyperplasia and damage	Absent	Mild less than 5% pyknotic	Moderate less than 10% pyknotic	Severe more than 10% pyknotic
Perivascular and peribronchiloar cuffing	Absent	Mild The cuffs average 2–3 cells in thickness	Moderate The leukocytes surrounding blood vessels are about 4–5 cells thick	Severe These cuffs average 6–15 cells in thickness
Area involved in disease	Absent	Mild less than 10%	Moderate Less than 20%	Severe More than 20%

### Sphingosine prevents infection of human lung tissue with mycobacteria

**Fig. 5:**
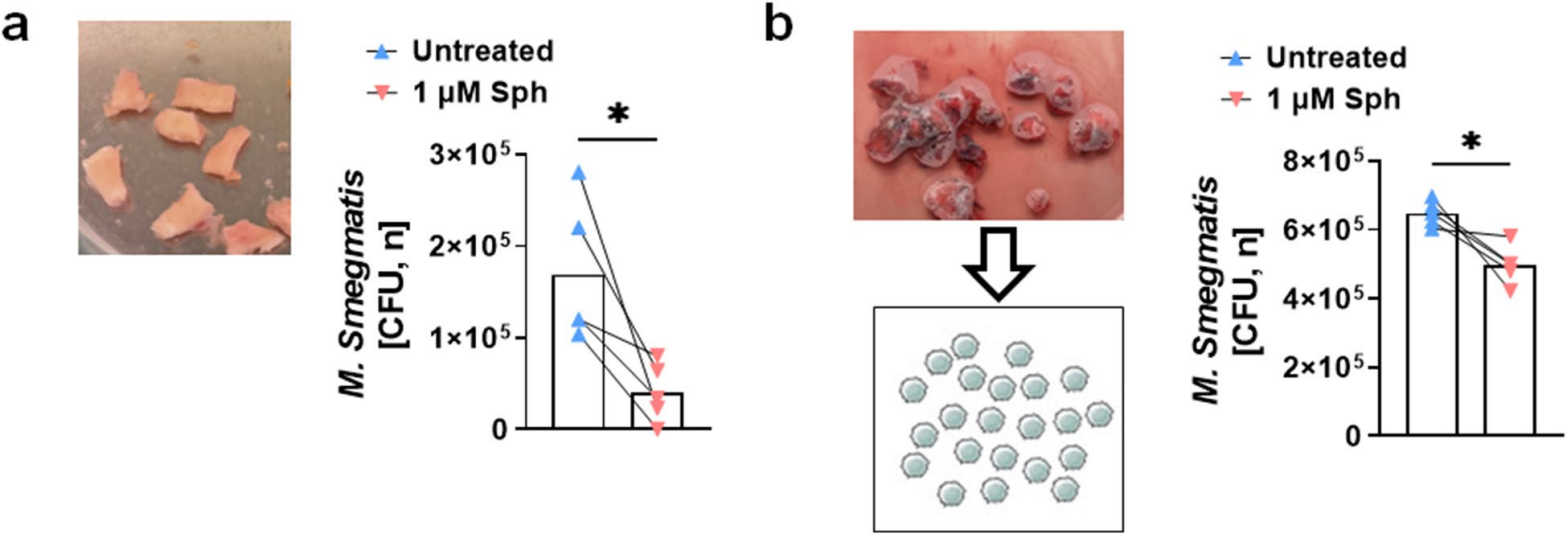
Sphingosine prevents infection of human lung tissue with mycobacteria. Patient bronchus (**a**) or lung parenchyma (**b**) was cut into small pieces, infected with *M. smegmatis*, and left untreated or treated with sphingosine. Infection was terminated after 4 h; tissue pieces were homogenized, and bacterial infection was determined by CFU assays. Shown are means ± SD of CFU numbers, n = 5. *p < 0.05, *t*-test

To investigate the anti-mycobacterial activity of sphingosine for mycobacterial infections in human tissues, we obtained bronchial and lung parenchyma samples from lung transplant patients, i.e. 3 patients with interstitial pulmonary fibrosis and 2 patients with chronic obstructive pulmonary disease. Bronchial samples were cut into small pieces, and their surfaces were infected with *M. smegmatis* and treated with sphingosine. The CFU results were normalized by the protein concentration of the bronchial samples. Remarkably, sphingosine treatment reduced bacterial numbers in human bronchi by almost 90% (Fig. 5a). Furthermore, we isolated single cells from lung parenchyma, infected them with *M. smegmatis,* and treated them with sphingosine or left them untreated. Again, we observed a reduction in bacterial burden after sphingosine treatment of lung parenchyma cells (Fig. 5b). These findings suggest that sphingosine exerts a promising anti-mycobacterial effect in reducing mycobacteria numbers in human lung tissue.

## Discussion

This study shows that sphingosine exerts marked bactericidal activity on various mycobacteria, i.e., *M. bovis* (BCG), *M. tuberculosis,* and *M. smegmatis*. Mechanistically, molecular studies showed that sphingosine disrupts the bacterial membrane, resulting in the release of ATP from mycobacteria. Our findings are in accordance with previously reported molecular mechanisms of sphingosine-mediated killing of *P. aeruginosa* and *S. aureus*[12]. Previous studies demonstrated that sphingosine binds to cardiolipin in *P. aeruginosa* and *S. aureus* by electrostatic attraction, which results in membrane alterations and membrane leakage. Mycobacteria abundantly express cardiolipin in their plasma membrane[24, 25]. Mycobacteria have a lipid bilayer outer membrane and an inner membrane, and cardiolipin resides in the inner membrane of the mycobacteria[26]. Sphingosine may be able to reach inner membranes of mycobacteria, because sphingosine forms micelles that may be able to cross the outer bacterial membrane. However, since the membranes of, for instance *P. aeruginosa*, *S. aureus* or *E. coli* are very different from those of *BCG* or *M. tuberculosis*, it is not possible to translate findings on non-tuberculous bacteria to BCG or *M. tuberculosis*. In fact, *M. tuberculosis* and BCG require higher concentrations of sphingosine for elimination, a phenomenon also observed with many antibiotics[27–29] used in mycobacterial treatment. We assume that this is caused by the highly hydrophobic capsule of mycobacteria, which may impede sphingosine’s access. Thus, the effective concentration of sphingosine on/in the mycobacteria might be significantly lower.

It has been previously shown that sphingosine induces membrane permeabilization in bacteria[12]. In detail, it has been demonstrated that a protonated NH_2_ group of sphingosine is required for its anti-bactericidal effect. The protonated NH_2_ group of sphingosine binds to bacterial cardiolipin, which seems to be required for the bactericidal effect of sphingosine. In accordance, *P. aeruginosa* and *E. coli* mutants deficient for enzymes of the cardiolipin synthesis pathway are resistant to sphingosine. Cardiolipin is absent from mammalian plasma membranes and only mitochondrial membranes contain cardiolipin. A specific effect of sphingosine on a bacterial lipid is also consistent with the findings that mammalian membranes of epithelial cells in the respiratory tract are resistant to inhalation of sphingosine at concentrations between 10 μM up to 500 μM. Inhalation of even high doses of sphingosine did not have any detectable toxic effects on epithelial cells of the respiratory tract in mice and even more important in isolated perfused and ventilated pigs, in which the sphingosine as inhaled via a tracheal catheter into the bronchia. A simple detergent-like effect would be very likely incompatible with these in vivo data.

The electron microscopy data also shows that sphingosine does not simply lyse the bacteria but rather induces the formation of membranes rolls and bleb-like structures. It is important to note that at this time, the bacteria are already killed. It remains to be determined whether these changes are part of the bactericidal effect of sphingosine or an attempt of the bacteria to repair the membrane, similar to the exclusion of membrane-incorporated toxins.

Furthermore, our electron microscopy studies found rapid changes in electron dense intracellular material.However future studies are required to define the nature of these changes and whether these changes are primary effects of sphingosine or secondary to membrane alterations induced by sphingosine.

Our in vivo data show that sphingosine inhalation exerts a remarkable effect on the number of BCG in the lung in a sub-acute model. Our findings suggest that sphingosine kills planktonic bacteria in the lung upon inhalation, probably by mechanisms similar to those in vitro. However, it may be that sphingosine in addition also reaches intracellular bacteria and even bacteria in granuloma. Further sphingosine may alter the activity of macrophages, antigen-presenting cells, or lymphocytes in vivo to promote the killing of mycobacteria in the lung. Separate studies that are beyond the focus of the present study are needed to determine whether sphingosine, directly or indirectly, also affects mycobacteria in granuloma.

Sphingosine is a natural lipid mediator, and its use in treatment could be considered safer than other treatments of *M. tuberculosis* for patients due to its endogenous nature. Compared to current TB treatments, which can cause inflammation and pulmonary injury, sphingosine's may have less side effects, at least based on the available mouse, pig and human ex vivo data. However, our data are the 1st study to show the effect of sphingosine on M. tuberculosis infections in vivo. The studies need to be extended, and it needs to be investigated, for instance, whether the bacteria develop resistance mechanisms to sphingosine if they are exposed several times, whether sphingosine has side effects if used for longer times, and whether sphingosine is still active in truly chronic infections.

Our studies demonstrate that sphingosine kills mycobacteria, including *M. tuberculosis* and *M. smegmatis*, and even greatly reduces the infection of mouse lungs with BCG. Sphingosine also prevents infection of human ex vivo bronchi with mycobacteria. Sphingosine maintained its activity after application as an aerosol.This finding suggests that sphingosine may serve as a novel drug for treating pulmonary mycobacterial infection.

## Methods

### Human samples

All experiments on human samples were performed with the permission of the Ethics Committee of the University Hospital Essen, permission number 17–7326-BO.

Freshly removed bronchial pieces and lung parenchyma from human lung transplant tissue were immediately cut into similarly sized pieces (10 × 10 × 10 mm), washed three times in Dulbecco's Modified Eagle Medium (DMEM), and placed in a 24-well plate for infection with 3.5 × 10^5^ M*. smegmatis*, prepared as described below, in 500 µL DMEM.

Lung parenchyma tissues were transferred into a petri dish and cut into small pieces with scissors. The tissues were then transferred into a 50-mL Falcon tube with ice-cold phosphate-buffered saline (PBS) to wash away remaining blood on the tissues, and the PBS was removed by passing the tissue through a 40-µm cell strainer. The tissues on the top of the strainer were transferred to a new Falcon tube containing 0.6 mg/mL collagenase (Merck, Darmstadt, Germany; # C2-22-BC), 0.02 mg/mL Elastase (Serva, Heidelberg, Germany; #20,930.01), and 17 units/mL DNase (QIAGEN, Hilden, Germany; # 79254) for 30 min at 37 °C. Samples were then washed in 10% fetal calf serum (FCS) in PBS, and the cell suspension was passed through a 70-µm strainer to a 15-mL Falcon tube for centrifugation at 300 × g for 5 min at 4 °C. The cell pellets were resuspended in 2 mL of red blood cell lysis buffer (Biolegend, San Diego, CA, USA) for 2 min at room temperature, followed by the addition of 10 mL RPMI-1640/10% FCS and pelleted again by centrifugation. Cells were resuspended in RPMI-1640 medium (with HEPES buffer), counted by trypan blue staining, and seeded at 1 × 10^4^ cells per mL in 96-well plates for infection.

For both bronchial and parenchymal samples, *M. smegmatis* was prepared as below and used for infection. After 4 h of infection, the non-internalized bacteria were extensively washed off, and fresh medium containing 1 µM sphingosine or medium containing 0.025% OGP was added for 1 h. Samples were washed, and cells were lysed by adding saponin to a final concentration of 0.5% for 15 min to release intracellular bacteria. The lysates were diluted and plated on LB agar plates. Colonies were counted after 3 days of incubation at 37 °C.

### Mouse experiments

Procedures performed on the animals were in accordance with the North Rhine–Westphalia (NRW) State Office for Nature, Environment and Consumer Protection (LANUV), Recklinghausen, Germany, permission number: AZ 81–02.04.2019.A148.

We used wild-type mice on a C57BL/6 background. Mice were housed and bred in the mouse facilities of the University Hospital, University of Duisburg-Essen, Germany, and the University of Cincinnati (Cincinnati, OH, USA). They were tested for common murine pathogens according to the 2002 recommendations of the Federation of European Laboratory Animal Science Associations (FELASA), and they were kept free of pathogens. All experiments were performed according to the FELASA regulations and the Animal Research: Reporting of In Vivo Experiments (ARRIVE) guidelines.

### Mycobacteria preparation

A green fluorescent protein (GFP)-expressing BCG (GFP-BCG) strain was used for both in vivo and in vitro infections. The GFP-BCG strain was constructed by transforming BCG with the dual reporter plasmid pSMT3L × EGFP. For the infection experiments, bacteria were cultured and shaken at 120 rpm at 37 °C in Erlenmeyer flasks containing 10 mL Middlebrook 7H9 Broth supplemented with glycerol (BD Biosciences, Heidelberg, Germany) supplemented with 50 µg/mL hygromycin B for maintaining GFP plasmids. After 5 to 7 days of incubation, the bacteria were used for experiments. *M. smegmatis* (ATCC19420) was prepared as above and used for experiments after 16 to 24 h growth. *M. tuberculosis* (ATCC 27294, H37Rv) was cultured in Mycobacteria Growth Indicator Tubes with PANTA supplement (Becton–Dickinson Microbiology System, Sparks, MD, USA) at 37 °C for 5 to 7 days.

GFP-BCG and *M. smegmatis* were finally collected by centrifugation at 3000 × g for 10 min. The bacterial pellet was resuspended in PBS buffer (pH 7.4) and vortexed for 5 min. Additionally, GFP-BCG was passed through a syringe with a 25-gauge needle for 10 times to avoid clumps. The bacteria were then bath-sonicated for 5 min at 4 °C to suspend any remaining clumps. Unseparated bacterial clumps were finally removed by centrifugation at 220 × g for 2 min. The supernatant containing single GFP-BCG or *M. smegmatis* was carefully collected. We calculated the bacterial counts on the basis of the optical density (OD) by using an Eppendorf (Hamburg, Germany) BioPhotometer 6131 (OD_600_ 1 = 10^8^ bacteria).

*M. tuberculosis* (ATCC 27294, H37Rv) was collected when the bacteria reached logarithm growth, transferred into a Falcon tube, and sonicated for 5 min. Remaining clumps were allowed to sediment for 10 min, the supernatant containing single bacteria was carefully collected, and the bacterial number was calculated on the basis of the OD as measured with an Eppendorf Biophotometer 6131.

### Macrophage preparation

For preparation of BMDMs, mice were put to death, and femurs and tibias were rinsed with minimal essential medium (MEM; Gibco, Paisley, UK) supplemented with 10% FCS (Gibco), 10 mM HEPES (Roth GmbH, Karlsruhe, Germany; pH 7.4), 2 mM l-glutamine, 1 mM sodium pyruvate, 100 μM nonessential amino acids, 100 U/mL penicillin, and 100 μg/mL streptomycin (Gibco). The isolated cells were flushed through a 23G needle for obtaining single cells. The isolated cells were washed, and 3 to 6 × 10^6^ cells were cultured in Petri dishes in MEM containing 20% L-cell supernatant as a source of macrophage colony-stimulating factor (M-CSF). Fresh MEM/L-cell supernatant medium was applied to the culture every 3 days. Macrophages matured within the next 6 days and were used on day 8 of culture. Cells were grown at 37 °C in 5% CO_2_.

For alveolar macrophages, mice were put to death, the trachea was exposed and catheterized with a polyethylene tube, and bronchoalveolar lavage was performed with a total of 15 mL ice-cold PBS. The fluid was collected and centrifuged for 5 min at 300 × g at 4 °C. The pellets containing alveolar macrophages were resuspended in MEM/HEPES, counted, and then seeded for further experiments.

### Infection and treatment

For in vitro assays, BMDMs or alveolar macrophages were seeded in 24- or 96-well plates, left uninfected, or infected with GFP-BCG in MEM/10 mM HEPES (pH 7.4) at a bacteria-to-host cell ratio (multiplicity of infection, MOI) of 1:1 to 10:1 for a specified indicated time. Synchronous infection conditions and enhanced interactions between bacteria and host cells were achieved by centrifuging (55 × g) the bacteria onto the cells for 5 min. The end of centrifugation was defined as the starting point of infection.

For in vivo infections, bacteria were prepared as described above and then pelleted at 3,000 × g for 10 min. BCG bacteria were resuspended in 0.9% NaCl, and 5 × 10^4^ CFU bacteria in 50 µL were intranasally infected into mice. After 3 weeks of infection, the mice were left untreated or were treated with 0.9% NaCl or 125 µM sphingosine (dissolved in 0.9% NaCl) twice a day for one week by inhalation in a Buxco Inhalation Tower (Data Science International, New Brighton, MN, USA). At the end of the infection and treatment, the mice were put to death by cervical dislocation, blood was collected, lungs were removed, and the right upper lobe was separated for counting CFUs. The remaining lung tissue was fixed in 4% paraformaldehyde (PFA) and embedded in paraffin after serial dehydration with an ethanol-to-xylol gradient, or embedded in Tissue-Tec (Sakura Finetek USA, Torrance, CA, USA) and shock-frozen in liquid nitrogen.

### Sphingosine quantification by liquid chromatography–tandem mass spectrometry (LC–MS/MS)

Samples of cell culture media were filled with water to 1 mL (if necessary) followed by the addition of 110 μL 10 × Baker buffer (300 mM citric acid, 400 mM disodium hydrogen phosphate, pH 3.0). For lipid extraction, 2 mL 1-butanol and 1 mL water-saturated 1-butanol were added. The extraction solvent contained sphingosine-d_7_ (Sph-d_7_) (Avanti Polar Lipids, Alabaster, AL, USA) as an internal standard. Extraction was facilitated by intensive vortexing (1500 rpm) for 10 min at room temperature. Afterwards, samples were centrifuged for 5 min at 2200* g* (4 °C). The upper organic phase was dried under reduced pressure with a Savant SpeedVac concentrator (Thermo Fisher Scientific, Dreieich, Germany). Dried residues were reconstituted in 200 μL acetonitrile/methanol/water (47.5:47.5:5 v:v:v; 0.1% formic acid) and subjected to LC–MS/MS sphingosine quantification as described^41^. The instrumentation used consisted of a 1260 Infinity liquid chromatography (LC) system equipped with a Poroshell 120 EC-C8 column (3.0 × 150 mm, 2.7 μm) coupled to a 6490 triple-quadrupole mass spectrometer (all from Agilent Technologies, Waldbronn, Germany) operating in positive electrospray ionization (ESI +) mode. The mass transitions *m/z* 300.3 → 282.3 (252.3) for sphingosine (Sph) and *m/z* 307.3 → 289.3 (259.3) for Sph-d_7_ were recorded (qualifier product ions in parentheses). Sph was directly quantified via its deuterated internal standard Sph-d_7_ (0.25 pmol on column). Data evaluation was performed with MassHunter Quantitative Analysis software (Agilent Technologies).

### Colony forming assay

To study bacterial killing by macrophages, we seeded cells in 96-well plates (1 × 10^4^ per well) and infected them with a bacteria-to-host cell ratio (MOI) of 1:1 for 4 h. The cells were washed and left untreated or treated with sphingosine for a specified time. At the end of infection, the cells were washed and lysed by incubation in 0.5% saponin at 37 °C for 15 min. The lysates were diluted and plated out on 7H10 agar plates, and colonies were counted after 21 days of incubation at 37 °C.

To count CFUs in the lungs of infected mice, the right upper lobe was collected and transferred to a 6-well plate, cut into small pieces, and homogenized with a scalpel and the plunger of a 5 mL syringe in 2 mL of 0.5% saponin for 15 min of incubation at 37 °C. The lysates were diluted with PBS and plated on 7H10 agar plates. The bacteria were grown for 21 days at 37 °C, and colonies were counted.

### Immunofluorescence and hematoxylin & eosin staining

Macrophages were grown on coverslips and were left uninfected or were infected with GFP-BCG. The infected cells were left untreated or were treated with sphingosine as described above. At the end of infection, cells were washed, fixed in 1% PFA (Sigma) in PBS (pH 7.4) for 10 min, and washed three times with PBS. For immunofluorescence staining, macrophages were permeabilized with 0.1% Triton X-100/PBS for 5 min at room temperature, washed with PBS, and incubated for 1 h with 5% FCS (Thermo Fisher Scientific, Waltham, MA, USA) to block nonspecific binding. The samples were then washed three times with PBS and were mounted with Mowiol (Kuraray Specialities Europe GmbH, Okayama, Japan) or DAPI (Abcam, Cambridge, UK).

For hematoxylin and eosin (H&E) staining, paraffin-embedded lung samples were sectioned at 6 µm, dewaxed, rehydrated, and stained with Mayer’s hemalaun solution (Roth, Karlsruhe, Germany; #T865.1) for 5 min. Samples were rinsed with water for 15 min, stained with 1% eosin solution for 2 min, and washed again with water. Stained sections were dehydrated with ethanol to xylene and embedded in Eukitt mounting medium (Sigma-Aldrich). Specimens were examined with a Leica DMi8 transmitted light microscope with a 40 × lens using identical settings. All images were analyzed with ImageJ software (Fiji, Wisconsin, USA).

### Direct treatment of mycobacteria with sphingosine

Sphingosine (Avanti Polar Lipids, Alabaster, AL, USA; #860490P) was resuspended in 7.5% OGP at 20 mM or 100 mM concentration as a stock. Sphingosine was sonicated (Bandelin Sonorex, Berlin, Germany) for 10 min before each use and diluted in 0.0075% OGP or in 0.9% NaCl to 1 to 100 µM.

Bacteria were diluted in PBS to a concentration of 1 × 10^6^ CFU/mL for GFP-BCG and *M. smegmatis*, or of 1 × 10^5^ CFU/mL for *M. tuberculosis*. Bacteria were incubated with sphingosine for up to 24 h for BCG and *M. smegmatis* or up to 3 weeks for *M. tuberculosis* at 37 °C.

For determination of CFU, BCG and *M. smegmatis* were diluted and plated in 7H10 agar plates, and colonies were counted after incubation for 14 days (for BCG) or 3 days (for *M. smegmatis*) at 37 °C. For *M. tuberculosis*, growth was observed by BacTec MGIT automated mycobacterial detection system (BD) every 1 to 3 days for up to 21 days.

For membrane permeability, control or sphingosine-treated BCG were incubated with 100 nM TO-PRO-3 iodide (Life Technologies, Inc., Arcadia, CA, USA) for 10 min, pelleted, and analyzed by flow cytometry with Attune NxT (Life Technologies, Inc.). *M. tuberculosis* was incubated with OGP or sphingosine for 10 min, followed by centrifugation at 3,000 × g for 10 min. The pelleted bacteria were resuspended with 100 µL zombie violet (1:100 dilution; Biolegend) and incubated for 15 min. Bacteria were washed with PBS, pelleted, fixed with 4% PFA for 15 min, and washed with PBS for flow cytometry analysis.

For measuring the remaining ATP, control or sphingosine-treated BCG were pelleted and resuspended with BacTiter-Glo (Promega, Madison, WI, USA) according to the manufacturer’s instructions and were measured with a luminescence reader.

### Transmission electron microscopy

Samples were fixed with 2.5% glutaraldehyde in 0.1 M sodium cacodylate buffer (pH 7.4) overnight at 4 °C. The samples were postfixed with 1% osmium tetroxide plus potassium ferrocyanide 1% in 0.1 M sodium cacodylate buffer for 1 h at 4°. After three water washes, samples were dehydrated in a graded ethanol series and embedded in an epoxy resin (Sigma-Aldrich). Ultrathin Sects. (60–70 nm) were obtained with an Ultratome Leica Ultracut EM UC7 ultramicrotome, counterstained with uranyl acetate and lead citrate, and viewed with a Tecnai G^2^ (FEI) transmission electron microscope operating at 100 kV. Images were captured with a Veleta (Olympus Soft Imaging System, Muenster, Germany) digital camera.

### Cell viability assay

To study the viability of the macrophages upon sphingosine treatment, we seeded 1 × 10^4^ cells per well in a 96-well plate. The cells were left untreated or were treated with various concentrations of sphingosine for specified times. The viability of the cells was measured by CellTiter 96 assay (Promega). Briefly, cells were washed after incubation and resuspended in tetrazolium containing medium for 4 h. The plate was analyzed by a plate reader at 570 nm.

### Statistical analysis and quantification

All data were obtained from independent experiments and presented as arithmetic mean ± standard deviation (SD); *n* represents the number of independent experiments or mice. Student's *t*-test for single comparisons and ANOVA for multiple comparisons were used to detect statistically significant differences; statistical significance was set at the level of p ≤ 0.05. If appropriate, data were quantified with ImageJ or Flowjo. GraphPad Prism 9 statistical software (GraphPad Software, La Jolla, CA, USA) was used for statistical analysis and graphical presentations.

## Supplementary Information

Below is the link to the electronic supplementary material.

ESM 1 Sphingosine-1-phosphate does not kill mycobacteria in vitro. Bacillus Calmette–Guérin (BCG) was treated with sphingosine-1-phosphate (S1P) at the indicated concentrations for 24 h. Bacteria were plated on agar plates, and colony-forming units (CFUs) were counted as a measurement of the survival of BCG after S1P treatment. Shown are mean ± SD, *n*=3. *p<0.05, **p<0.01, ***p<0.001, ****p<0.0001, one-way ANOVA. OGP, n-Octyl-β-D-glucopyranoside; S1P, sphingosine-1-phosphate (PNG 409 KB)

High Resolution Image (TIF 59.8 KB)

## Data Availability

All original data are available upon request.
