# Supplementary figures and images for: Sphingosine kills Mycobacteria and suppresses mycobacterial lung infections
Source: J Mol Med (Berl). 2025 Mar 28;103(5):547–58. doi: 10.1007/s00109-025-02534-z (PMC12078450; doi:10.1007/s00109-025-02534-z)

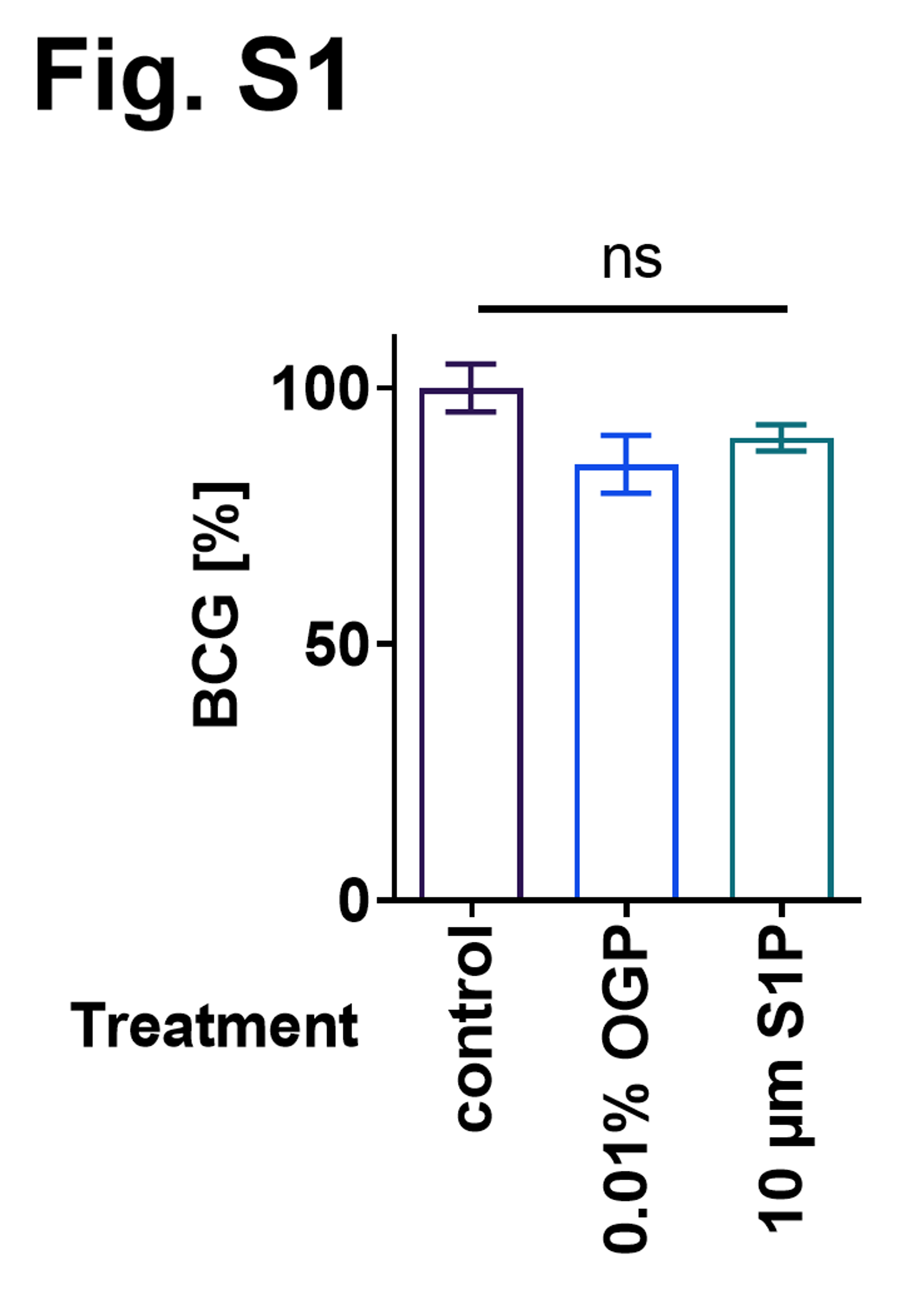

Supplement: Supplementary file 1 — Sphingosine-1-phosphate does not kill mycobacteria in vitro. Bacillus Calmette–Guérin (BCG) was treated with sphingosine-1-phosphate (S1P) at the indicated concentrations for 24 h. Bacteria were plated on agar plates, and colony-forming units (CFUs) were counted as a measurement of the survival of BCG after S1P treatment. Shown are mean ± SD, n=3. *p<0.05, **p<0.01, ***p<0.001, ****p<0.0001, one-way ANOVA. OGP, n-Octyl-β-D-glucopyranoside; S1P, sphingosine-1-phosphate (PNG 409 KB) [file 109_2025_2534_Fig6_ESM.png]

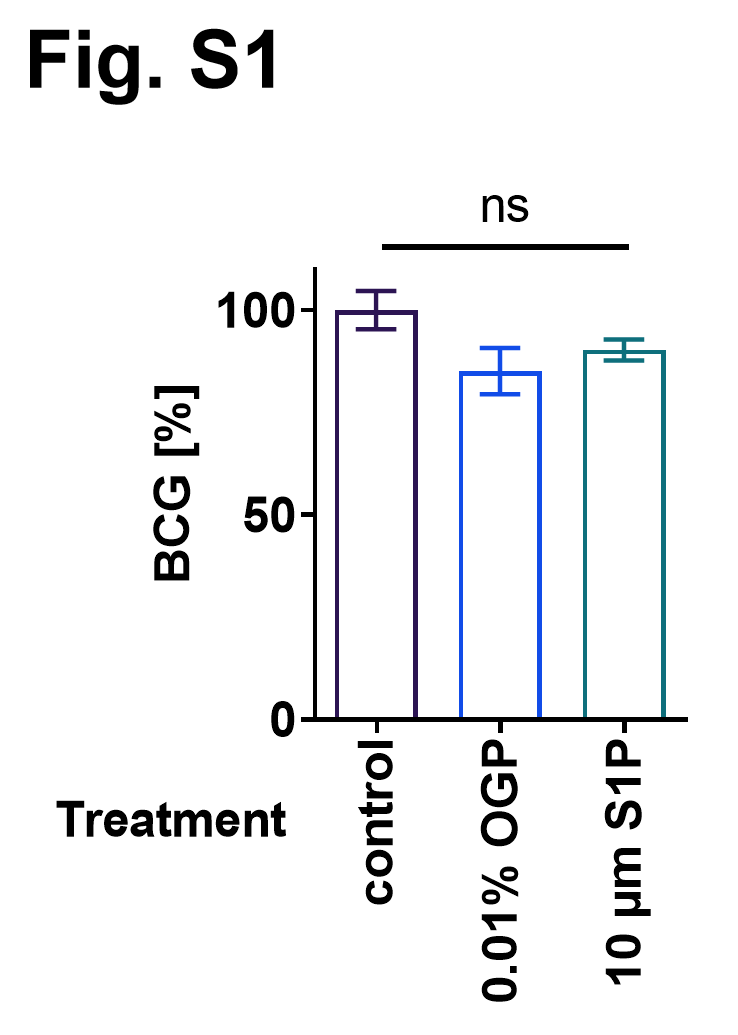

Supplement: Supplementary file 2 — High Resolution Image (TIF 59.8 KB) [file 109_2025_2534_MOESM1_ESM.tif]
